# Assessment of nephrotoxicity following lutetium-177 PSMA I&T radioligand therapy: a comparative study with docetaxel chemotherapy

**DOI:** 10.1007/s00259-026-07890-7

**Published:** 2026-04-16

**Authors:** Florian P Kirchhoff, Lisa Steinhelfer, Christian H. Pfob, Constantin Lapa, Philipp E. Hartrampf, Andreas K. Buck, Robert Tauber, Hannah Schäfer, Christoph Schmaderer, Cornelia Fütterer, Bernhard Haller, Matthias Jahnen, Karina Knorr, Jürgen E. Gschwend, Wolfgang A. Weber, Matthias Eiber, Lukas Lunger

**Affiliations:** 1 Department of Urology, School of Medicine, Technical University of Munich, TUM University Hospital, Ismaninger Str. 22, 81675 Munich, Germany; 2 Department of Nuclear Medicine, School of Medicine and Health, Technical University of Munich, TUM University Hospital, Munich, Germany; 3 Department of Diagnostic and Interventional Neuroradiology, School of Medicine and Health, Technical University of Munich, TUM University Hospital, Munich, Germany; 4 Faculty of Medicine, University of Augsburg, Nuclear Medicine, Augsburg, Germany; 5 Department of Nuclear Medicine, University Hospital Wurzburg, Wurzburg, Germany; 6 Department of Nephrology, School of Medicine and Health, Technical University of Munich, TUM University Hospital, Munich, Germany; 7 School of Medicine and Health, Institute of AI and Informatics in Medicine, Technical University of Munich, TUM University Hospital, Munich, Germany

**Keywords:** Nephrotoxicity, PSMA-Lutetium I&T, Radioligand therapy, mCRPC, Docetaxel

## Abstract

**Abstract:**

To compare changes in estimated glomerular filtration rate (eGFR) following Lutetium-177 prostate-specific membrane antigen I&T radioligand therapy (^177^Lu-PSMA I&T) and docetaxel chemotherapy in metastatic castration-resistant prostate cancer (mCRPC).

**Methods:**

This retrospective multicenter analysis included 145 patients treated with ^177^Lu-PSMA I&T and 39 patients treated with docetaxel. Inclusion required ≥ 4 cycles of ^177^Lu-PSMA I&T or ≥ 10 docetaxel administrations and ≥ 12 months of eGFR follow-up. Docetaxel patients were excluded if ^177^Lu-PSMA I&T was initiated within 12 months after therapy initiation. Patients were stratified by treatment sequence (docetaxel only, pre-chemo and post-chemo ^177^Lu-PSMA I&T). Linear mixed models (LMM) assessed longitudinal eGFR changes with adjustment and matching for baseline renal risk factors (arterial hypertension, diabetes mellitus, age ≥ 65 years, prior platinum-based chemotherapy).

**Results:**

Median age (interquartile range, IQR) was 75.0 (67.0–77.0) years in the docetaxel group, 81.5 (77.5–87.0) years in the pre-chemo ^177^Lu-PSMA I&T subgroup (*n* = 58), and 75.5 (71.0–78.3) years in the post-chemo ^177^Lu-PSMA I&T subgroup (*n* = 87). Mean eGFR declined over time in both pre- and post-chemo ^177^Lu-PSMA I&T subgroups compared with docetaxel-treated patients, with significant differences emerging earlier in the pre-chemo subgroup (from 3 months) and later in the post-chemo subgroup (from 12 months), persisting through follow-up. At 12 months, severe eGFR decline (≥ 30%) was more frequent after ^177^Lu-PSMA I&T than docetaxel (pre-chemo: 32.8% vs. 2.6%, OR = 18.5; post-chemo: 31.0% vs. 2.6%, OR = 17.1; both *p* < 0.001). LMM confirmed a significant time-dependent eGFR decline with ¹⁷⁷Lu-PSMA I&T versus docetaxel (entire cohort β=-1.56; pre-chemo β=-1.41; both *p* < 0.001).

**Conclusion:**

^177^Lu-PSMA I&T is associated with a gradual, time-dependent decline in renal function compared with docetaxel, independent of prior chemotherapy exposure and baseline renal risk factors. These findings highlight the importance of baseline renal assessment and longitudinal kidney function monitoring, particularly as ^177^Lu-PSMA RLT is increasingly considered earlier in the treatment course.

## Introduction

Lutetium-177 prostate-specific membrane antigen (^177^Lu-PSMA) radioligand therapy (^177^Lu-PSMA I&T) is an established standard therapy in metastatic castration-resistant prostate cancer (mCRPC) following disease progression on androgen-receptor–pathway inhibitors and taxane chemotherapy [1, 2]. Given its observed efficacy and preferable toxicity profile in later stage mCRPC, ongoing trials investigate the use of ^177^Lu-PSMA radioligand therapy in earlier mCRPC stages [3–5] or even metastatic hormone sensitive prostate cancer (mHSPC) [6]. Nevertheless, several reports have raised concern about the medium to long-term effects of ^177^Lu-PSMA radioligand therapy on renal function. In a recent retrospective study involving 106 patients who underwent ^177^Lu-PSMA I&T radioligand therapy (^177^Lu-PSMA I&T) at three German tertiary referral centers, a significant decline of at least 15% in estimated glomerular filtration rate (eGFR) in 45% of patients 12 months post-treatment compared to their baseline values was observed [7]. Among those, nearly half experienced severe (≥ 30% - <40%) and very severe (≥ 40%) reductions. However, due to its retrospective design, a limited number of patients with follow-up data beyond 12 months, the lack of a control group and the complex clinical context with comorbidities, risk factors, and prior therapies potentially impacting renal function, the data should be interpreted with caution [7, 8].

Such effects might have limited clinical significance in late stage mCRPC. However, in light of recent results from the PSMAfore and SPLASH trials with a potential early use of ^177^Lu-PSMA radioligand therapy prior to docetaxel chemotherapy in patients with longer life-expectancies makes a renewed evaluation of its long-term toxicity profile, especially nephrotoxicity, highly relevant [3, 4].

Therefore, the aim of this retrospective multicenter study was to further expand on previous findings and investigate renal function over time in mCRPC patients treated with ^177^Lu-PSMA I&T as compared to a matched control group of mCRPC patients receiving docetaxel.

## Materials and methods

### Patients

#### Study cohort

For this trial, the databases of three German tertiary referral centers (Technical University of Munich, University of Wuerzburg, and University of Augsburg) were reviewed to identify additional patients and follow-up data beyond the 106 previously reported between December 2015 to September 2023 [7].

**Fig. 1:**
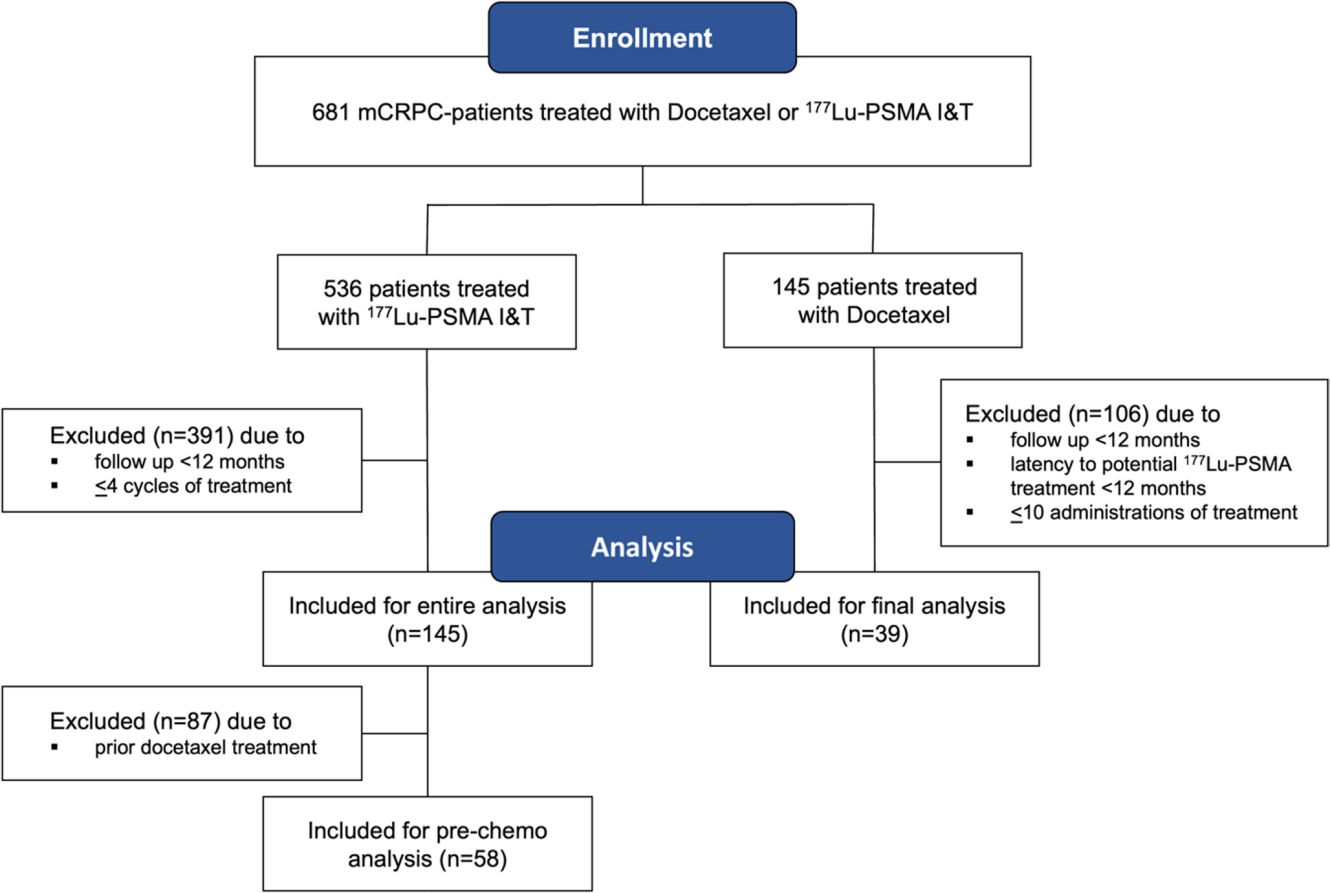
Recruitment flow chart

**Fig. 2:**
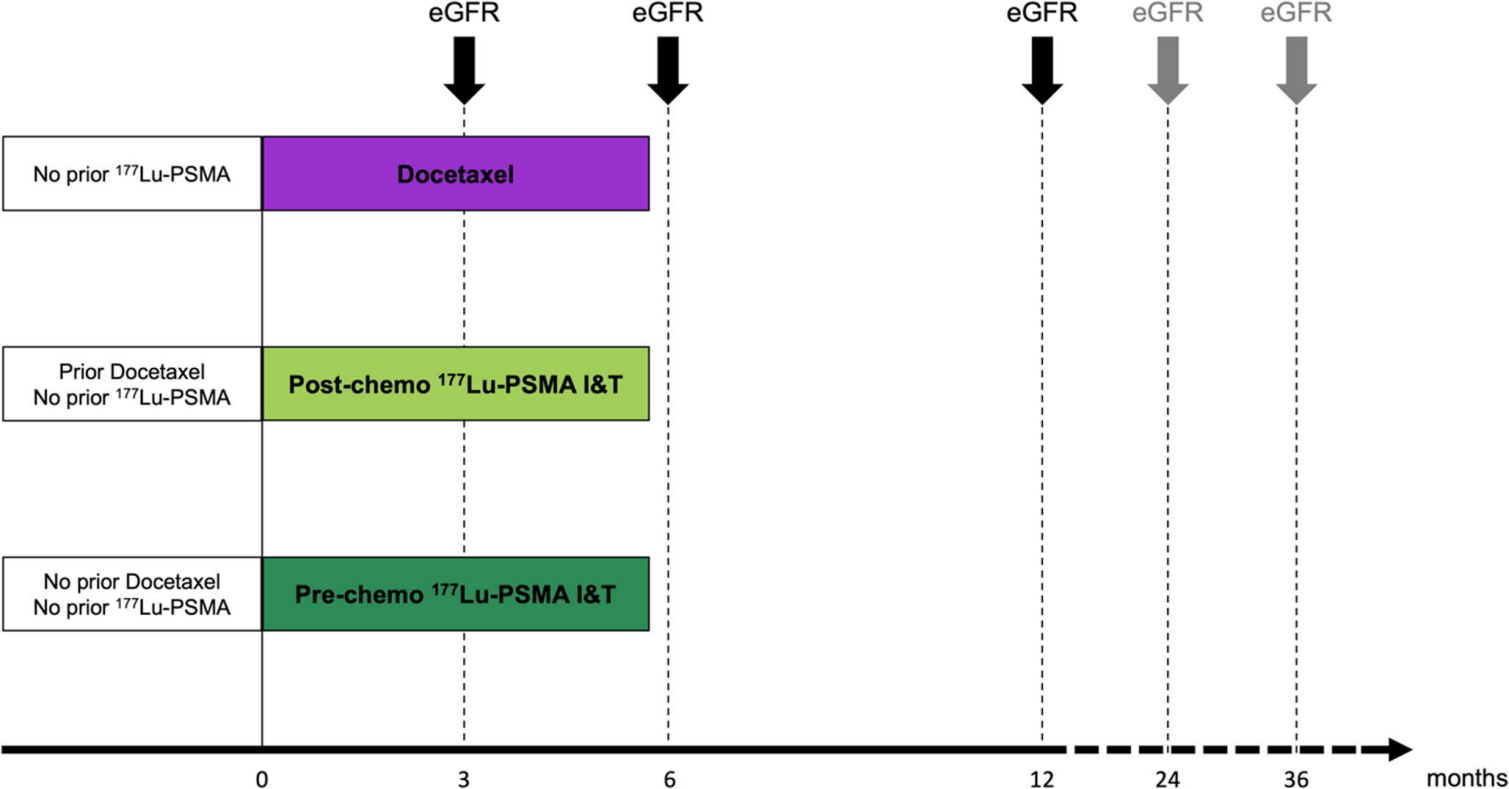
Schematic illustration depicting the chronological course of patient management, including the timing of docetaxel and ^177^Lu-PSMA I&T administration and longitudinal renal function assessments. *Abbreviations*: eGFR = estimated glomerular filtration rate; ^177^Lu = Lutetium 177; PSMA = prostate specific membrane antigen

The inclusion criteria for the study cohort (^177^Lu-PSMA I&T) required patients to have completed a minimum of four cycles of ^177^Lu-PSMA I&T, had at least one year of follow-up eGFR data post-treatment initiation, and showed no evidence of urinary tract obstruction based on pre-treatment Technetium-99 m (99mTc)-MAG3 scintigraphy. Prior docetaxel therapy was allowed. To investigate eGFR dynamics, the ^177^Lu-PSMA I&T group was stratified into patients having received prior docetaxel therapy (post-chemo) and docetaxel-naïve patients (pre-chemo), in line with the approach of the PSMAfore and SPLASH trials [3, 4]. The latter analysis excluded patients who had received prior docetaxel chemotherapy. The recruitment flow chart is illustrated in Fig. 1. Chronological course of patient management, including the timing of docetaxel and ^177^Lu-PSMA I&T administration, and longitudinal renal function assessments is depicted in Fig. 2.

All patients received the ^177^Lu-PSMA I&T ligand. The institutional eligibility criteria for employing ^177^Lu-PSMA I&T as an individual treatment decision were previously outlined and published [9]. The production and administration of ^177^Lu-PSMA I&T adhered to the German pharmaceutical law (Arzneimittelgesetz, § 13.2b) and complied with the standards set by the responsible regulatory bodies. Informed consent was obtained from all patients, and treatments were conducted in accordance with the declaration of Helsinki article 37, addressing “unproven interventions in clinical practice.” Prior to ^177^Lu-PSMA I&T treatment, each patient received intravenous hydration (500 mL 0.9% NaCl) starting 30 min before the procedure, followed by intravenous administration of ^177^Lu-PSMA I&T.

#### Control group

To establish a control group, the institutional database at the Technical University of Munich was screened to identify patients with mCRPC who underwent docetaxel chemotherapy without prior ^177^Lu-PSMA I&T between January 2015 and July 2023. These patients had to meet the following inclusion criteria: at least ten administrations of docetaxel, a minimum of one year of eGFR follow-up post-treatment initiation, and a latency of at least one year before potentially undergoing ^177^Lu-PSMA I&T. For patients who received docetaxel and subsequently underwent ^177^Lu-PSMA I&T after the required latency period for this analysis (12 months), eGFR follow-up values were only included in the analysis of eGFR dynamics for the docetaxel group until the start of ^177^Lu-PSMA I&T treatment. All eligible patients during this period were screened consecutively; the number of patients screened and included is detailed in the study flow chart (Fig. 1).

Docetaxel was administered every 2 weeks at a dose of 50 mg/square meter body surface (maximum: 100 mg), with a maximum of 12 administrations.

Approval for the retrospective analysis was obtained from the local institutional review boards (references 115/18S, 2020-40, 20200609 01).

### Measures

Throughout a minimum of 12 months from therapy initiation, all serum creatinine values were recorded to calculate the estimated glomerular filtration rate using the Chronic Kidney Disease Epidemiology Collaboration (CKD-EPI) formula for every patient. The CKD-EPI formula, incorporating variables such as serum creatinine levels (Scr), age, gender, and ethnicity, is represented as eGFR = 141 × min(Scr/κ, 1)^α^ × max(Scr/κ, 1)^−1.209^ × 0.993^Age^, with Scr representing serum creatinine, κ set at 0.9, and α at -0.411. The “min” function indicates the minimum of Scr/κ or 1, and “max” denotes the maximum of Scr/κ or 1 [10]. A monoexponential curve was fitted to each patient’s eGFR data from treatment initiation to at least 12 months, providing standardized eGFR estimates at predefined time points (3, 6, 12, 24 months).

Renal failure stages, as defined by CKD-EPI criteria, were determined at both baseline and the 12-month follow-up for all patients. Percent changes from baseline were computed at specified time points (3, 6, 12, 24 months) for each patient, with clinically relevant %-eGFR decrease cut-offs (≥ 15% - <30% (moderate), ≥ 30% - <40% (severe) and ≥ 40% (very severe) established in previous studies [11, 12].

The following risk factors potentially associated with impaired renal function were recorded at baseline: arterial hypertension (defined as use of specific antihypertensive drugs), diabetes mellitus (characterized by HbA1c levels ≥ 6% or the use of antidiabetic medication), age ≥ 65 years and prior platinum-based chemotherapy [7, 13].

Docetaxel exposure was reported as the number of documented administrations. This approach was chosen because definitions of a “docetaxel cycle” vary across centers, with some institutions defining one cycle as two administrations (e.g. day 1 and day 15 within a 28-day interval), whereas others refer to each individual administration as one cycle. Accordingly, in this manuscript docetaxel treatment is reported as the number of administrations documented in the source data.

### Statistical analyses

Continuous patient characteristics are described with mean and standard deviation (SD) for normally distributed values and with median and interquartile ranges (IQR) for non-parametric data. Categorical variables are presented as with absolute frequencies and percentages. Follow-up duration was defined based on the availability of serum creatinine measurements, with the last recorded value determining the end of follow-up.

At various timepoints, we compared eGFR values between ^177^Lu-PSMA I&T subgroups and docetaxel chemotherapy using Mann-Whitney-U-Tests and Wilcoxon-Rank-Sum-Tests, means and standard deviations (SD) are provided. Chi-square tests were calculated to investigate the association of the categorical eGFR decrease from baseline (< 30) versus an at least severe decrease (≥ 30%) with the type of treatment (^177^Lu-PSMA I&T (pre/post-chemo) versus docetaxel).

Linear mixed models (LMM) were used to model eGFR in dependence of the fixed effects therapy at baseline (^177^Lu-PSMA I&T (all) versus docetaxel (reference)), time (within the docetaxel reference group), the interaction of therapy (^177^Lu-PSMA I&T) and time, number of present risk factors at baseline, number of treatment lines and overall cycles. To account for the different distribution of renal risk factors between the therapy groups at baseline and their significant impact on renal function as postulated in a previous study [7], we performed a 2:1 matching process based on previously described risk factors.

For the pre-chemo subgroup (^177^Lu-PSMA I&T without prior docetaxel), another LMM was calculated using the same variables as previously described, following a 1:1 matching process (due to the low number of matching candidates for the ^177^Lu-PSMA I&T without docetaxel treatment) for the same nephrotoxic risk factors. In all models, a random intercept for each patient was considered. For the mixed models, we report the estimated regression coefficients (β), 95% confidence intervals (CIs), and corresponding p-values for the various covariates. P-values < 0.05 were considered statistically significant (two-sided test).

Data analyses were conducted using SPSS 26 (IBM, Armonk, New York, USA) and R version 4.4.2 (R Core Team (2023). R: A language and environment for statistical computing. R Foundation for Statistical Computing. Vienna, Austria, https://www.R-project.org/). Illustrations were also created using R version 4.4.2.

## Results

### Patient characteristics

Recruitment flow chart is illustrated in Fig. 1. Patient characteristics are presented in Table 1. A total of 184 mCRPC patients fulfilled the inclusion criteria. Among them, 145 patients (39 new patients, 106 patients of the previous study [7] with extended follow up data) underwent ^177^Lu-PSMA I&T, while 39 patients received docetaxel chemotherapy. The median (IQR) baseline eGFR was 74 (65–92) ml/min in the pre-chemo ^177^Lu-PSMA I&T group, 84 (72–94) ml/min in the post-chemo ^177^Lu-PSMA I&T group and 84 (63–93) ml/min in the docetaxel group. At baseline, impaired renal function (eGFR ≤ 60 ml/min) was observed in 17% (10/58) of patients in the pre-chemo ^177^Lu-PSMA I&T cohort, 16% (14/87) of patients in the post-chemo ^177^Lu-PSMA I&T cohort and 13% (5/39) of the patients undergoing docetaxel chemotherapy. The median (range) follow-up duration was 12.7 months (12.0–58.5).

**Table 1:** Patient characteristics and treatment comparison of Docetaxel, pre-chemo and post-chemo177Lu-PSMA I&T (*n* = 184)

	Docetaxel (*n* = 39)	Pre-chemo ^177^Lu-PSMA I&T (*n* = 58)	Post-chemo ^177^Lu-PSMA I&T (*n* = 87)
Age (years)			
Median (IQR)	75.0 (67.0–77.0)	81.5 (77.5–87.0)	75.5 (71.0–78.3)
Baseline eGFR [ml/min]			
Median (IQR)	83.8 (62.9–92.9)	74.2 (64.8–92.2)	84.1 (71.7–93.9)
Prior treatment lines (no. pts., (%))			
0	0 (0)	2 (3)	6 (7)
1	30 (77)	22 (38)	25 (29)
2	8 (20)	30 (52)	35 (40)
3	1 (3)	4 (7)	19 (22)
* ≥* 4	0 (0)	0 (0)	2 (2)
No. of cycles/administrations			
Median no. (IQR)	12 (12–12)	6 (5–8)	6 (5–8)
< 6 (^177^Lu-PSMA I&T) (%)		19 (33)	26 (30)
≥ 6 (^177^Lu-PSMA I&T) (%)		39 (67)	61 (70)
Risk factors* at baseline (no. pts., (%))			
0	4 (10)	3 (5)	4 (5)
1	14 (36)	15 (26)	17 (20)
2	18 (46)	25 (43)	40 (46)
3	3 (8)	14 (24)	22 (25)
4	0 (0)	1 (2)	4 (5)

*Risk factors: arterial hypertension, diabetes mellitus, age ≥ 65 years, prior platinum-based chemotherapy

*eGFR* estimated glomerular filtration rate, *IQR* interquartile range, ^177^Lu = Lutetium 177; no. = number, *PSMA* prostate specific membrane antigen, *pts* patients

### Comparison of eGFR changes over time between treatment groups

**Fig. 3:**
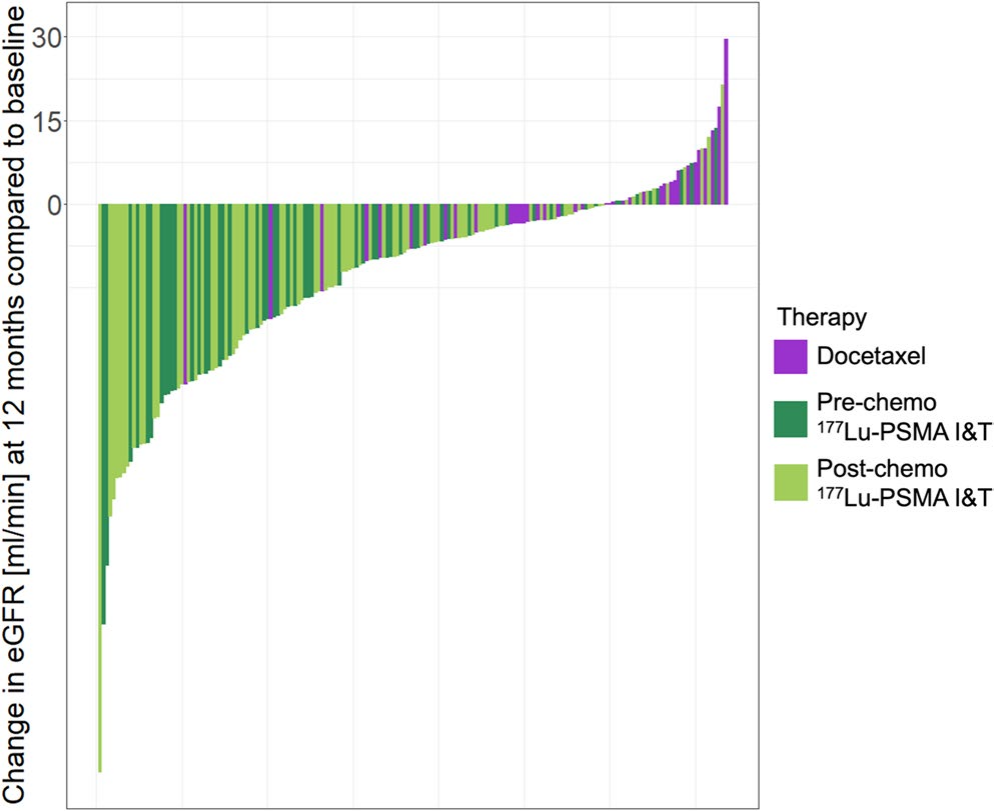
Waterfall plot illustrating individual total changes in eGFR [ml/min] at 12 months after therapy initiation compared to baseline. Violet = docetaxel-only; dark green = pre-chemo ¹⁷⁷Lu-PSMA I&T; medium green = post-chemo ¹⁷⁷Lu-PSMA I&T. Abbreviations: eGFR = estimated glomerular filtration rate; ^177^Lu = Lutetium 177; PSMA = prostate specific membrane antigen

**Fig. 4:**
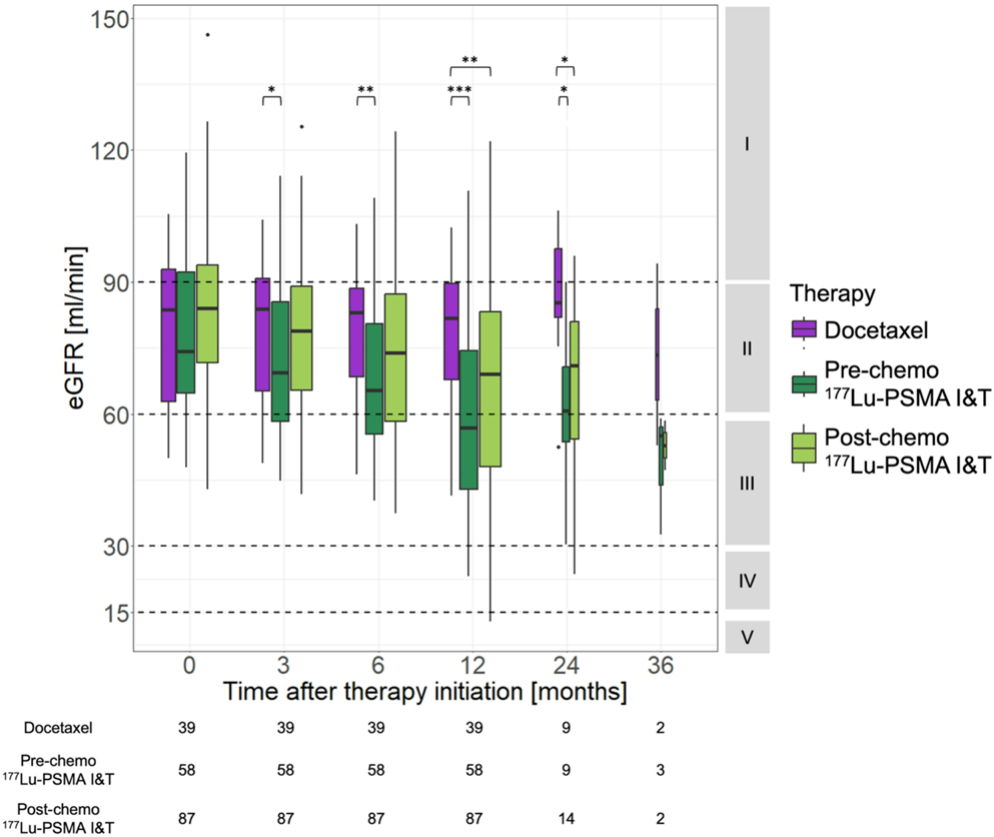
Boxplots illustrating estimated glomerular filtration rate (eGFR) at baseline and at 3, 6, 12, 24, and 36 months after therapy initiation, stratified by treatment sequence: docetaxel-only (control cohort), ^177^Lu-PSMA I&T after prior docetaxel exposure (post-chemo ^177^Lu-PSMA) I&T, and ^177^Lu-PSMA I&T without prior docetaxel exposure (pre-chemo ^177^LuPSMA I&T). Dashed horizontal lines indicate thresholds of chronic kidney disease stages (labels shown on the right). Brackets denote the number of patients contributing data at each time point (docetaxel / pre-chemo / post-chemo ^177^Lu-PSMA I&T). Box width is proportional to sample size. P-values refer to pairwise between-group comparisons at individual time points, with docetaxel serving as the reference group. Significance levels are indicated as follows: * *p* < 0.05; **p < 0.01; ***p < 0.001. Colors: violet = docetaxel-only; dark green = pre-chemo ^177^Lu-PSMA I&T; medium green = post-chemo ¹⁷⁷Lu-PSMA I&T. Abbreviations: eGFR = estimated glomerular filtration rate; ^177^Lu = lutetium-177; PSMA = prostate-specific membrane antigen. The figure is descriptive, primary inference regarding longitudinal treatment effects is based on linear mixed-effects models

Individual total changes in eGFR from baseline within 12 months after therapy initiation are depicted in Fig. 3. Figure 4 shows longitudinal eGFR distributions according to treatment sequence. Renal function in docetaxel-only patients remained largely unchanged over follow-up, whereas patients treated with ^177^Lu-PSMA I&T demonstrated a gradual downward shift in eGFR over time. Differences between docetaxel-only and ^177^Lu-PSMA I&T-treated patients became statistically apparent by 3 months in the pre-chemo ^177^Lu-PSMA I&T and 12 months in the post-chemo ^177^Lu-PSMA I&T subgroup and were observed through 24 months, with the most pronounced separation at 12 months (pre-chemo ^177^Lu-PSMA I&T vs. docetaxel: 3 months: 70 (IQR 58–86) ml/min vs. 84 (65–91) ml/min, *p* = 0.036; 6 months: 65 (56–80) ml/min vs. 83 (68–89) ml/min, *p* = 0.002; 12 months: 57 (43–74) ml/min vs. 82 (68–90) ml/min, *p* < 0.001; 24 months: 61 (54–71) ml/min vs. 85 (82–98) ml/min, *p* = 0.031; post-chemo ^177^Lu-PSMA I&T vs. docetaxel: 12 months: 69 (48–83) ml/min vs. 82 (68–90) ml/min, *p* = 0.003, 24 months: 71 (54–81) ml/min vs. 85 (82–98) ml/min, *p* = 0.028). At later follow-up intervals, variability increased and fewer patients contributed data; however, the relative ordering of treatment groups remained consistent. No significant differences were observed at baseline as well as at any timepoint between the two ^177^Lu-PSMA I&T subgroups.

**Fig. 5:**
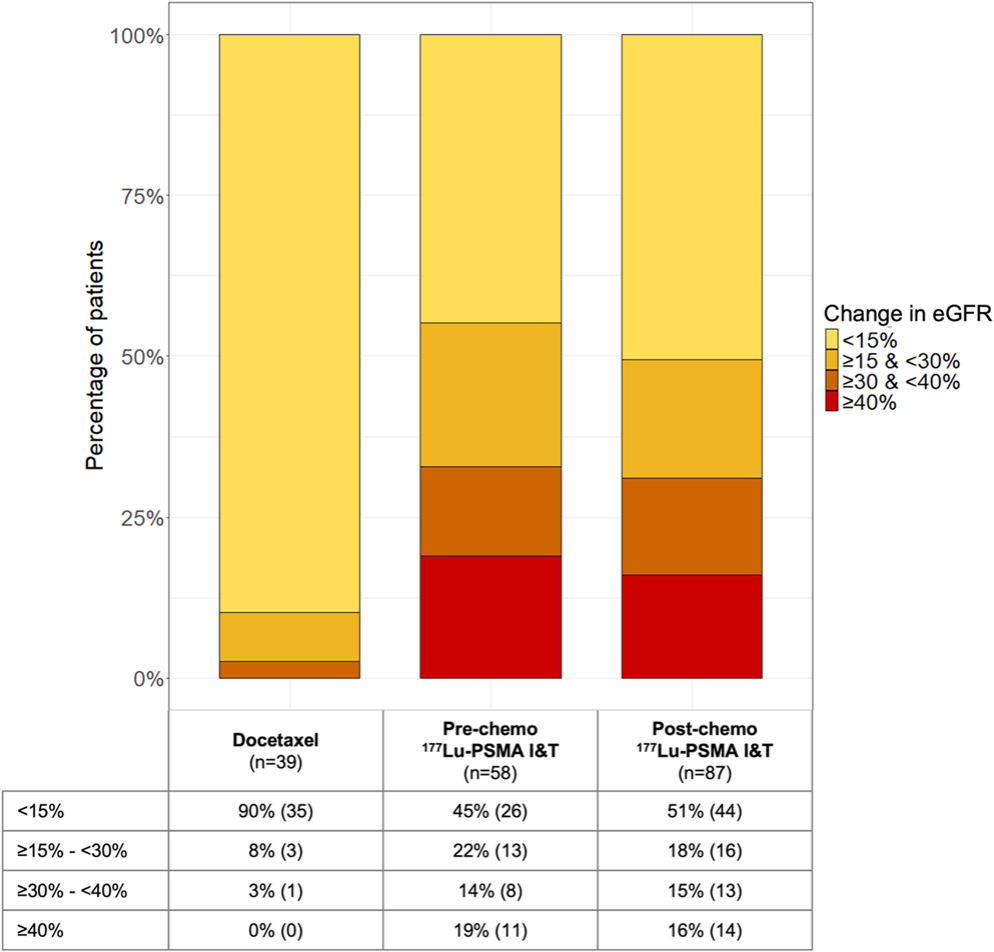
Proportion of patients with eGFR decrease at 12 months after therapy initiation (% of total, no. pts.)

At 12 months, the distribution of eGFR changes differed significantly between treatment groups. Severe renal impairment (eGFR decline ≥ 30%) was uncommon in docetaxel-treated patients (2.6%, 1/39), whereas it occurred substantially more frequently in patients treated with ^177^Lu-PSMA I&T, both in the pre-chemo (32.8%, 19/58) and post-chemo (31.0%, 27/87) subgroups (OR = 18.5 and OR = 17.1, *p* < 0.001, respectively). Very severe eGFR decline (≥ 40%) was observed exclusively in the ^177^Lu-PSMA I&T groups and not in the docetaxel cohort (Fig. 5).

The LMM (entire study cohort, 2:1 matching) analysis demonstrated that treatment with ^177^Lu-PSMA I&T was associated with a stronger eGFR decline over time than for treatment with docetaxel (β_Interaction_ = -1.56, 95% CI -1.81 to -1.30, *p* < 0.001). For docetaxel treatment, no significant association between time and eGFR was observed (β = -0.08, 95% CI -0.27 to 0.11, *p* = 0.426). The number of risk factors present at baseline (β = -7.88, 95% CI -12.09 to -3.66, *p* < 0.001) was strongly linked to a reduced eGFR across all time points. In contrast, the number of prior treatment lines did not significantly impact eGFR (β = 3.21, 95% CI -0.53 to 6.95, *p* = 0.092). (Table 2)

**Table 2:** Linear mixed model for the association of eGFR with therapy at baseline (177Lu-PSMA I&T; reference = docetaxel), time (within the docetaxel reference group), the interaction of therapy (177Lu-PSMA I&T) and time (Therapy×Time) indicating the difference in eGFR development over time between treatment groups, number of risk factors at baseline*, number of treatment lines and overall cycles of 177Lu-PSMA I&T after performing a 2:1-matching for risk factors in the entire study cohort (177Lu-PSMA I&T: *n* = 77, docetaxel: *n* = 39)

Variable	Estimates	95% CI	*p*
	76.98	57.31–96.65	< 0.001
Time	-0.08	− 0.27–0.11	0.426
Therapy [Ref: Docetaxel]	2.04	-9.31–13.38	0.725
Therapy×Time	-1.56	-1.81 – -1.30	**< 0.001**
Risk factors	-7.88	-12.09 – -3.66	**< 0.001**
Treatmentlines	3.21	-0.53–6.95	0.092
Cycles overall	0.90	-0.67–2.47	0.261

Note: conditional R²: 0.856. CI = confidence interval for estimates

*eGFR* estimated glomerular filtration rate, ^177^Lu = Lutetium 177, *PSMA* prostate specific membrane antigen

*Risk factors associated with renal impairment: arterial hypertension, diabetes mellitus, age ≥ 65 years, prior platinum-based chemotherapy

The LLM analysis for the pre-chemo cohort after 1:1 matching for risk factors (^177^Lu-PSMA I&T: *n* = 38, docetaxel: *n* = 38) was consistent with the LLM results for the entire study cohort. First, a significantly stronger decline in kidney function over time was observed for^177^Lu-PSMA I&T compared to docetaxel (β_Interaction_ = -1.41, 95% CI -1.65 to -1.17, *p* < 0.001), while no significant change over time was observed for the docetaxel group (β = -0.08, 95% CI -0.24 to 0.07, *p* = 0.291). Additionally, a greater number of risk factors at baseline was associated with worse kidney function across all time points (β = -6.85, 95% CI -11.68 to -2.02, *p* = 0.006). However, neither the number of previous treatment lines, nor the number of treatment cycles significantly affected the observed kidney function across all time points (β = -2.25, 95% CI -8.70 to 4.20, *p* = 0.493 and β = 1.14, 95% CI -0.52 to 2.80, *p* = 0.179, respectively). (Table 3)

**Table 3:** Linear mixed model for the association of eGFR with therapy at baseline (177Lu-PSMA I&T; reference = docetaxel), time (within the docetaxel reference group), the interaction of therapy (177Lu-PSMA I&T) and time (Therapy×Time) indicating the difference in eGFR development over time between treatment groups, number of risk factors at baseline*, number of treatment lines and overall cycles of 177Lu-PSMA I&T in the pre-chemo subgroup and following 1:1-matching for risk factors (177Lu-PSMA I&T: *n* = 38, docetaxel: *n* = 38)

Variable	Estimates	95% CI	*p*
	79.41	58.24–100.58	< 0.001
Time	-0.08	− 0.24–0.07	0.291
Therapy [Ref: Docetaxel]	6.17	-5.75–18.09	0.309
Therapy×Time	-1.41	-1.65 – -1.17	**< 0.001**
Risk factors	-6.85	-11.68 – -2.02	**0.006**
Treatmentlines	-2.25	-8.70–4.20	0.493
Cycles overall	1.14	-0.52–2.80	0.179

Note: conditional R²: 0.887. CI = confidence interval for estimates

*eGFR* estimated glomerular filtration rate, ^177^Lu = Lutetium 177, *PSMA* prostate specific membrane antigen

*Risk factors associated with renal impairment: arterial hypertension, diabetes mellitus, age ≥ 65 years, prior platinum-based chemotherapy

## Discussion

Previous studies have raised concerns about the nephrotoxic effects of ^177^Lu-PSMA radioligand therapy in patients with mCRPC; two studies investigated ^177^Lu-PSMA-617, whereas one study used ^177^Lu-PSMA I&T [2, 7, 14]. This retrospective study extends these findings for ^177^Lu-PSMA I&T by introducing a control cohort of mCRPC patients with docetaxel chemotherapy, addressing limitations of earlier studies [8]. In addition to providing a comparative analysis with docetaxel-treated patients, this current analysis also presents data from an expanded ^177^Lu-PSMA I&T population, including a pre-chemo subgroup of patients who received ^177^Lu-PSMA I&T without prior docetaxel chemotherapy. This analysis allows for a more nuanced understanding of the nephrotoxic effects of ^177^Lu-PSMA I&T, and provides important insights into how renal function is affected by ^177^Lu-PSMA I&T across different treatment sequences.

First, in this study, a significant decline in eGFR in patients treated with ^177^Lu-PSMA I&T was observed compared to those who received docetaxel chemotherapy. As illustrated in Fig. 4, separation between docetaxel-treated patients and ^177^Lu-PSMA I&T-treated patients (depending on docetaxel pretreatment) became statistically apparent as early as 3 months after therapy initiation and was most pronounced at 12 months. While previous findings were limited by the lack of an adequate control group [7], the current comparative analysis supports and strengthens the hypothesis that kidney function gradually declines over time as a direct result of ^177^Lu-PSMA I&T and not as part of disease progression or aging. This effect is reflected in an odds ratio exceeding 17 for experiencing a severe eGFR decline (≥ 30%) at 12 months following initiation of ^177^Lu-PSMA I&T compared to docetaxel, regardless of prior docetaxel therapy.

Importantly, this effect seems to be driven by an impaired kidney function at baseline in context of the presence of nephrotoxic risk factors and undergoing ^177^Lu-PSMA I&T over time. This supports the hypothesis that nephrotoxic effects associated with ^177^Lu-PSMA I&T may unfold gradually over time and not immediately, as could be assumed for radiation nephropathy [15]. The delayed separation between treatment groups and the progressive nature of eGFR decline observed in the longitudinal analyses are consistent with a cumulative nephrotoxic effect that manifests over months rather than as an acute renal injury. In line with that, neither time within the docetaxel reference group significantly impacted kidney function over time, nor the number of prior treatment lines (as a surrogate for more advanced disease) demonstrated a significant effect on the baseline kidney function. Notably, baseline creatinine clearance values were comparable across treatment groups with overlapping interquartile ranges (Fig. 4/Table 1), indicating that differences in subsequent renal trajectories are unlikely to be explained by marked baseline renal impairment. Even after matching for risk factors at baseline, the analysis of the entire study cohort revealed that ^177^Lu-PSMA I&T treatment over time remained significantly associated with decreasing eGFR, suggesting that the nephrotoxic effects of this therapy that were observed in comparison to the control group were not solely driven by baseline differences in these patient characteristics.

Given the potential adoption of ^177^Lu-PSMA radioligand therapy in earlier treatment sequences, a pre-chemo subgroup analysis in line with the approach of the PSMAfore and SPLASH trials [3, 4], was conducted. Comparable to later stage patients, this subgroup analysis showed a significant effect of ^177^Lu-PSMA I&T over time on the eGFR decline with an estimated reduction of 1.4 ml/min/month, after adjusting for risk factors at baseline (Table 3). The presence of a significant decline in the pre-chemo ^177^Lu-PSMA I&T subgroup at an earlier timepoint further supports the concept of a cumulative treatment-associated effect rather than an interaction with prior cytotoxic therapy. This finding highlights that nephrotoxic effects may be present even when ^177^Lu-PSMA I&T is used in early stages of the disease, even in the absence of prior chemotherapy exposure. Importantly, no consistent recovery of eGFR toward baseline values was observed over follow-up in the ^177^Lu-PSMA I&T-treated cohorts. However, this observation must be interpreted in the context of a heavily pretreated and comorbid patient population, in whom renal functional reserve and capacity for recovery may already be limited. In such patients, even modest additional renal insults may not be followed by full recovery, independent of the underlying mechanism.

Recent trials investigating ^177^Lu-PSMA-targeted radioligand therapy in combination or sequence with docetaxel have demonstrated encouraging antitumor activity without a clear increase in reported toxicity [16]. However, these studies were conducted in biologically and clinically distinct populations and were not designed to systematically assess longitudinal renal function trajectories. In addition, patients treated in earlier disease settings typically have greater baseline renal reserve and fewer cumulative nephrotoxic exposures than heavily pretreated mCRPC populations. However, the earlier separation in renal function observed in the docetaxel-naïve ^177^Lu-PSMA I&T subgroup in this study requires careful interpretation. Although eGFR distributions at baseline were largely overlapping across treatment groups in this analysis, docetaxel naïve patients treated with ^177^Lu-PSMA I&T tended to be older with a slightly lower baseline eGFR. During the period of patient recruitment, ^177^Lu-PSMA I&T was not yet an approved standard therapy and was therefore frequently considered for patients deemed less suitable/ineligible for cytotoxic chemotherapy. Against this background, the earlier emergence of renal function decline in the pre-chemo subgroup may reflect underlying patient selection and reduced baseline renal reserve rather than a fundamentally different nephrotoxic profile of ^177^Lu-PSMA I&T. Importantly, the consistent time-dependent association between ^177^Lu-PSMA I&T and declining eGFR across treatment sequences supports the robustness of the observed effect and underscores the need for careful renal monitoring irrespective of prior chemotherapy exposure. As such, the absence of increased toxicity in combination or sequencing studies does not preclude the possibility of gradual, time-dependent renal impairment in later-stage patients receiving PSMA radioligand therapy, underscoring the importance of dedicated renal monitoring.

Thus, despite the retrospective nature of this study and the limited number of patients, the clinical implications of our study are significant, particularly considering future clinical practice. Given the increasing life expectancy of mCRPC patients, considering a patient’s renal function and individual potential for nephrotoxic effects should be a critical consideration in treatment decisions between ^177^Lu-PSMA I&T and docetaxel, particularly following treatment with androgen receptor pathway inhibitors. The cumulative nature of renal function decline observed in this study underscores the importance of longitudinal monitoring of kidney function, especially in patients expected to receive prolonged or earlier exposure to ^177^Lu-PSMA I&T. Moreover, given that nephrotoxic effects of ^177^Lu-PSMA I&T unfold over time and ^177^Lu-PSMA I&T is frequently offered at large tertiary referral centers, but follow-up treatment may be decentralized, consistent monitoring of kidney function should be encouraged.

Several limitations of our study should be acknowledged. First, this analysis is based on retrospective data, including an expanded ^177^Lu-PSMA I&T cohort of previously reported patients (39 new patients, 106 patients of the previous study with extended follow up data). Second, although the dataset was increased, the follow-up period remains limited, which may be especially relevant for the use of ^177^Lu-PSMA I&T in patients with early stage mCRPC, which are expected to have a longer life expectancy. Third, our retrospective analysis lacked additional parameters for determining renal function (e.g. cystatin C) and we were unable to evaluate the impact of co-morbidities, complications of prostate cancer (e.g. infections) or late effects of other therapies. Fourth, this analysis includes patients treated with ^177^Lu-PSMA I&T. Since nephrotoxic effects may vary depending on the ligand used, these findings may not be directly transferable. Moreover, these findings may not apply to current therapeutic sequences or to the decision-making process between ^177^Lu-PSMA I&T and cabazitaxel chemotherapy. While the observed time-dependent decline in renal function is consistent with a cumulative treatment-associated effect, the retrospective design and the absence of dosimetry-based exposure metrics preclude definitive conclusions regarding a formal dose–response relationship. Furthermore, the ability to distinguish therapy-related irreversible renal injury from limited renal recovery in a morbid, pretreated population is inherently constrained in retrospective analyses. However, we believe that the addition of a docetaxel comparator group strengthens the evidence surrounding this topic and add to a critical gap in the field, especially due to the subgroup analysis in a pre-chemo scenario.

In conclusion, this study provides valuable insights into the directly therapy related nephrotoxic effect of ^177^Lu-PSMA I&T. By integrating longitudinal, treatment-sequence–specific renal function trajectories with comparative and matched analyses, the results demonstrate a gradual, time-dependent decline in renal function consistent with cumulative exposure. In the context of a pretreated and comorbid patient population, the absence of a clear signal of renal recovery suggests that observed renal impairment may be clinically relevant and potentially persistent, underscoring the importance of monitoring kidney function over time and the potential long-term nephrotoxic risks when selecting treatment regimens, especially with the introduction of ^177^Lu-PSMA I&T in earlier treatment sequences. Further prospective trials with larger patient cohorts are needed to confirm these findings and prospectively validate the suggested nephrotoxic risk factors and refine their impact on treatment strategies for mCRPC patients.

## Data Availability

The datasets generated and analyzed during the current study are available from the corresponding author on reasonable request.
